# Deep Sequencing the MicroRNA Transcriptome in Colorectal Cancer

**DOI:** 10.1371/journal.pone.0066165

**Published:** 2013-06-18

**Authors:** Kristina Schee, Susanne Lorenz, Merete Molton Worren, Clara-Cecilie Günther, Marit Holden, Eivind Hovig, Øystein Fodstad, Leonardo A. Meza-Zepeda, Kjersti Flatmark

**Affiliations:** 1 Department of Tumor Biology, Institute for Cancer Research, Clinic for Cancer, Surgery and Transplantation, The Norwegian Radium Hospital**,** Oslo University Hospital, Oslo, Norway; 2 Genomics Core Facility, Institute for Molecular Biosciences, University of Oslo, Oslo, Norway; 3 Institute for informatics, University of Oslo, Oslo, Norway; 4 Norwegian Computing Center, Oslo, Norway; 5 Bioinformatics Core Facility, Institute for Medical Informatics, Norwegian Radium Hospital, Oslo University Hospital, Oslo, Norway; 6 Department of Gastroenterological Surgery, Clinic for Cancer, Surgery and Transplantation, Norwegian Radium Hospital**,** Oslo University Hospital, Oslo, Norway; University of Aberdeen, United Kingdom

## Abstract

Colorectal cancer (CRC) is one of the leading causes of cancer related deaths and the search for prognostic biomarkers that might improve treatment decisions is warranted. MicroRNAs (miRNAs) are short non-coding RNA molecules involved in regulating gene expression and have been proposed as possible biomarkers in CRC. In order to characterize the miRNA transcriptome, a large cohort including 88 CRC tumors with long-term follow-up was deep sequenced. 523 mature miRNAs were expressed in our cohort, and they exhibited largely uniform expression patterns across tumor samples. Few associations were found between clinical parameters and miRNA expression, among them, low expression of miR-592 and high expression of miR-10b-5p and miR-615-3p were associated with tumors located in the right colon relative to the left colon and rectum. High expression of miR-615-3p was also associated with poorly differentiated tumors. No prognostic biomarker candidates for overall and metastasis-free survival were identified by applying the LASSO method in a Cox proportional hazards model or univariate Cox. Examination of the five most abundantly expressed miRNAs in the cohort (miR-10a-5p, miR-21-5p, miR-22-3p, miR-143-3p and miR-192-5p) revealed that their collective expression represented 54% of the detected miRNA sequences. Pathway analysis of the target genes regulated by the five most highly expressed miRNAs uncovered a significant number of genes involved in the CRC pathway, including APC, TGFβ and PI3K, thus suggesting that these miRNAs are relevant in CRC.

## Introduction

MicroRNAs (miRNA) are evolutionary conserved small (∼20–22 nt long), non-coding RNAs that regulate gene expression by binding to the 3′UTR of mRNA, thereby inhibiting translation [1]. They can bind with partial complementarity to mRNA to potentially downregulate several mRNAs. This makes the downstream studies somewhat challenging with multiple potential targets for each miRNA. Today there are approximately 1500 miRNAs annotated in the miRNA database (miRBase) [2] and it is estimated that up to 60% of protein coding genes may be regulated by miRNAs [3]. MiRNAs are essential for normal mammalian development and are involved in fine-tuning many biological processes, such as cell proliferation, differentiation, apoptosis and metabolism, and their involvement in cancer has sparked increased interest in miRNA biology [4]–[6]. They have been proposed as possible biomarkers because of their regulatory functions, chemical stability and the possibility of measuring miRNA in serum, plasma, stool and tissue samples [7]–[10].

Colorectal cancer (CRC) is one of the most common cancer forms in Western countries and a leading cause of cancer related deaths. It is a heterogeneous disease characterized by accumulation of genetic and epigenetic events, and influenced by lifestyle [11], [12]. Treatment decisions are still essentially based on the anatomical extent of disease at diagnosis, and the search for better biomarkers is warranted. MiRNAs have been examined for their potential role as diagnostic, prognostic and therapeutic biomarkers in CRC using hybridization based array technologies and quantitative RT-PCR (qRT-PCR) [13]–[16]. Using expression microarrays, expression of a large amount of pre-selected miRNAs can be detected, and the miRNA detection is based on signal intensities. Relative expression can be calculated using qRT-PCR, but when expanding to multiple parallel analyses, the number of miRNAs possible to analyze and RNA quantity may represent limitations. Deep sequencing has emerged as an attractive approach for global miRNA analysis, advantages including pooling of samples for high-throughput purposes, a wide detectable expression range, the ability to analyze expression of all annotated miRNAs and the possibility of detecting novel miRNAs.

In this work, deep sequencing was used to determine miRNA expression in 88 CRC tumor samples, and associations between expression levels and clinicopathological data and outcome were analyzed.

## Materials and Methods

### Patient Cohort and Sample Preparation

Between the years 1998–2000, 316 patients were recruited from five hospitals in the Oslo region [17], and prospectively included in the study at the time of primary surgery for assumed or verified colorectal cancer. The study was approved by the Regional Ethics Committee (Health Region II, Norway) and informed consent was obtained from the patients. Resected specimens were processed routinely for histopathological assessment at the time of surgery and classified according to the Tumor Node Metastasis (TNM) staging system. Sampling of additional tumor tissue was performed by the surgeon in the operating room after the specimen was removed from the patient, and the samples were immediately snap-frozen in liquid nitrogen. Samples were then transported to the research laboratory and kept for long-term storage at −80°C. The biopsies were sectioned using a cryostat microtome and hematoxylin-eosin stained slides were evaluated for tumor content by a pathologist (median tumor content in the samples was 50%, range 30–80%). The tumor tissue was then sliced into 10-µm sections using a cryostat microtome and stored at −80°C until RNA isolation. 120 cases were not included in the study for the following reasons: not invasive cancer (25), histology other than adenocarcinoma (5), distant metastasis at the time of surgery (34), preoperative chemoradiotherapy (2), inadequate surgical margins (7) and unknown stage of disease (1). In addition, frozen tissue samples were not obtainable in 46 cases. From the 196 samples in TNM stage I-III, 90 tumor samples were randomly selected for deep sequencing. After sequencing, two samples from the cohort were deemed degraded and were removed from further studies, leaving a sample cohort of 88 patients (Table 1). Follow-up data was obtained from the participating hospitals and from the general practitioners (for the patients not attending scheduled controls). Metastasis was verified by radiological examination and survival data was obtained from the National Registry of Norway and updated by October 1st 2008.

**Table 1:** Clinical and histopathological characteristics of the investigated patient cohort (n = 88).

**Sex**	Male	36
	Female	52
**TNM**	1	10
	2	51
	3	27
**pT**	1	2
	2	10
	3	70
	4	6
**pN**	0	61
	1	16
	2	11
**Tumor localization**	Right colon	33
	Transverse colon	5
	Left colon	24
	Rectum	26
**Differentiation**	Poor	10
	Intermediate	76
	Well	2
**Perinodal infiltration**	Absent	70
	Present	18
**Vascular invasion**	Absent	69
	Present	19
**Neural infiltration**	Absent	82
	Present	6

### RNA Isolation and Deep Sequencing

RNA was isolated from tumor tissue using TriReagent (Ambion Inc, TX) according to the manufacturer’s protocol and the total RNA concentration was measured by Nanodrop (ND-1000). The quality was assessed by the Agilent 2100 Bioanalyzer and samples with a RIN value of 7 and above were used for further analysis. Small RNA sequencing libraries were created following the Illumina®TruSeq™ Small RNA Sample Preparation protocol. In brief, 3` and 5` RNA adapter, specifically modified to target the ends of small RNA molecules, were ligated to 1 µg of high quality total RNA. Reverse transcription was performed to generate cDNA libraries and PCR was used to amplify and add unique index sequences to each library.

Small RNA libraries were pooled and 32 bases were sequenced for each cDNA molecule using an Illumina® Genome Analyzer IIx. Indexes were sequenced in order to identify the source of each read. The first run, containing 48 samples, was hampered by partially nonfunctional lanes in the flow cell and was therefore repeated. The data for run 1 and run 2 were combined for downstream expression analysis, as well as analyzed separately to determine the technical reproducibility of the experiments.

### Sequencing Data Analysis and Normalization

Real-time analysis, base calling and filtering of low quality reads were done by Illumina’s software packages (SCS2.9/RTA1.9 and Off-line Basecaller v1.9). Novoalign (V2.08.01 Novocraft 2010; www.novocraft.com) was used to cut remaining adapter sequence and map the reads to the reference human genome (hg19). All reads mapping to 10 or more genomic regions were excluded from further analysis. The mapped reads were annotated using known databases. The miRBase database release 18 (November 2011) was used to identify miRNAs, using BEDTools Version-2.16.2 [18]. The NCBI build “Homo_sapiens.NCBI.36.58” was used to identify other small RNA species and mRNA.

To calculate the read count for miRNAs, reads that mapped uniquely within a mature miRNA sequence with a maximum of one mismatch were considered hits. Reads mapping to more than one mature miRNA sequence were assigned according to the frequency of uniquely mapped reads found for these miRNAs. That means when two miRNAs shared a given number of multiple mapped reads, we identified the ratio of unique reads between these two miRNAs. This ratio was applied to divide the number of multiple mapped reads and assign them. If multiple hits were found to be perfectly mapped to one genomic region and mapped with mismatch to another one, only the perfect matches were considered.

For normalization of read counts, four different approaches were tested. We calculated the normalization factor for all samples by dividing the total number of reads, the number of reads aligned to the genome, allowing multiple hits or that map uniquely, or the number of reads mapped to annotated mature miRNAs with 1 million. The normalized expression values for each miRNA were generated by dividing the read count of the miRNA with the according normalization factor. After normalization, all miRNAs with read counts less than 10 across all patient samples were set to 0. The data set normalized against annotated mature miRNAs was chosen for the remaining analyses. Normalized and un-normalized read counts for all the samples have been made available at the Array Express website, accession number E-MTAB-1649 (http://www.ebi.ac.uk/arrayexpress/).

### Quantitative Real Time-PCR

qRT-PCR was previously performed for the entire cohort of 196 samples from which freshly frozen tissue was available to determine expression of six miRNAs: miR-21, miR-31, miR-92a, miR-101, miR-106a and miR-145 [19]. Briefly, cDNA synthesis and qRT-PCR were performed using TaqMan microRNA assays (Applied Biosystems, Foster City, CA) according to the manufacturer’s protocol. All samples were run in duplicates. Ct values for miRNAs were normalized against RNU44 and the relative expression was calculated using 2^−dCt^ method [20]. Results for these six miRNAs from the 88 samples that had been analyzed with both methods were used for comparison of data from deep sequencing with qRT-PCR. Associations between results from the deep sequencing and qRT-PCR were studied by linear regression analysis.

### Hierarchical Clustering and Statistical Analysis

Hierarchical clustering was performed to visualize expression patterns of all miRNAs. The normalized expression values were log2 transformed and unsupervised two-way hierarchical clustering was performed using Euclidean distance and weighted average linkage (WPGMA) to cluster miRNAs and samples simultaneously.

Two class unpaired significance analysis with multiple testing (10000 permutations) (SAM) [21] was used to identify miRNAs associated with clinicopathological parameters, using J-Express (2012 version) [22]. The input for SAM was normalized and log2 transformed and clinicopathological parameters were used as response variables. Ten thousand repeat permutations of the data were used to determine if the expression of miRNAs was significantly associated with one of the following clinicopathological parameters: TNM, pT, pN, tumor localization, differentiation, perinodal infiltration, vascular invasion and neural infiltration. The false discovery rate expressed as q-values less than 0.05 were considered statistically significant.

Overall and metastasis-free survival was calculated from date of surgery until date of death or diagnosis of metastasis. To identify miRNAs associated with overall and metastasis-free survival univariate Cox proportional hazard regression was applied to each miRNA, testing for associations with metastasis-free or overall survival. To account for multiple testing, adjusted p-values were calculated by controlling the false discovery rate (FDR), using the Benjamini-Hochberg procedure [23]. Then, for all the miRNAs simultaneously, the LASSO method in the Cox proportional hazards model [24], as implemented previously [25], was used to discover a set of miRNAs associated with the endpoints. “In the LASSO analysis no miRNAs were selected. No miRNAs were significant in the univariate Cox analysis after correction for multiple testing. In this last analysis, seven miRNAs (miR-339-5p, miR-7-1-3p, miR-365b-3p, miR-454-3p, miR-194-3p, miR-15b-3p and hsa-miR-4461) had p-value below 0.01 with metastasis development as endpoint, while three miRNAs (miR-101-5p, hsa-miR-873-5p and hsa-miR-3144-3p) had p-value below 0.01 with endpoint overall survival. All these ten miRNAs were expressed at very low levels in our cohort with the highest median expression observed for miR-454-3p with 187 reads to the lowest for miR-873-3p with 0 reads.

### Pathway Analysis for Target Genes of the Five Most Highly Expressed miRNAs

To investigate the biological influence of the most highly expressed miRNAs, target genes were identified using TarBase 6.0 [26]. This database contains target genes that have experimental support in addition to sequence-based prediction. The gene identities were uploaded into the web-based DAVID functional annotation tool for pathway analysis using the KEGG database [27], [28].

## Results

### Small RNA Sequencing and Annotation

**Figure 1:**
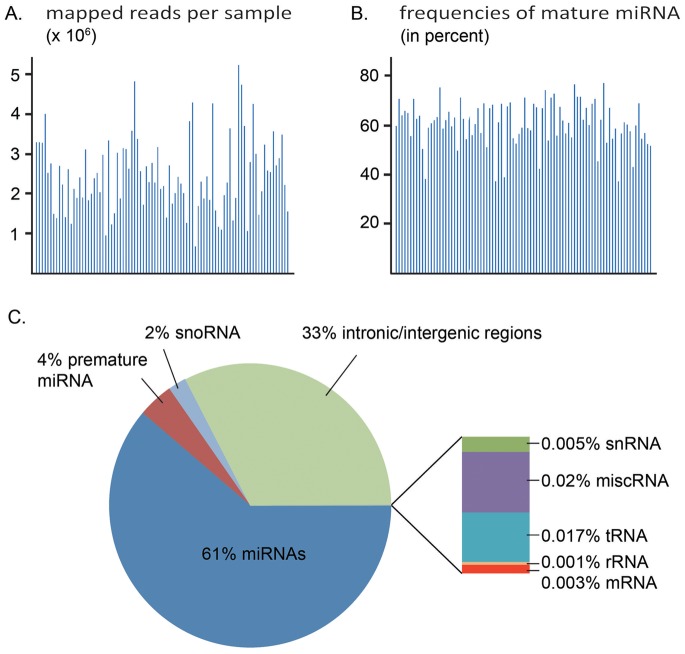
Overview of mapped reads, miRNAs and frequencies of RNA classes. **A.** Number of reads (x10^6^) mapped to the human genome (hg19) for all samples. These include all RNA species (premature miRNA, snoRNA, snRNA, miscRNA, rRNA, tRNA and mRNA).The sequences that did not match known sequence were matched against databases of intergenic and intronic regions of the human genome. **B.** Frequencies of reads mapped to annotated mature miRNAs for all samples using the microRNA database (miRBase release 18). **C.** Pie-chart representing percentages of the different RNA classes found in the data set.

The length of the detected sequences varied between 13 and 29 nucleotides after removal of the adapter sequence (longer reads were removed). The main portion of reads, 97.9%, was between 19 and 23 bases. In average, 2.6 million reads mapping to the human genome were obtained per tumor sample (Figure 1A). We identified the frequencies of reads falling into different classes of small RNA or other genomic regions and calculated the median frequencies comparing all 88 tumor samples. The frequency of reads mapping to mature miRNAs ranged from 37 to 77% in the libraries and gave a median of 61% (Figure 1B). For intronic/intergenic regions a median read frequency of 33% was found, and for premature miRNAs and snoRNAs, the median read frequencies were 4% and 2%, respectively (Figure 1C). In addition a small fraction of reads mapped to snRNA, miscRNA, tRNA, rRNA, and mRNA, together comprising a frequency of ∼0.05%. MiRNAs with less than 10 reads across all patient samples were considered not expressed. In total, 523 miRNAs were expressed in the data set.

### Normalization and Technical Replicates

**Figure 2:**
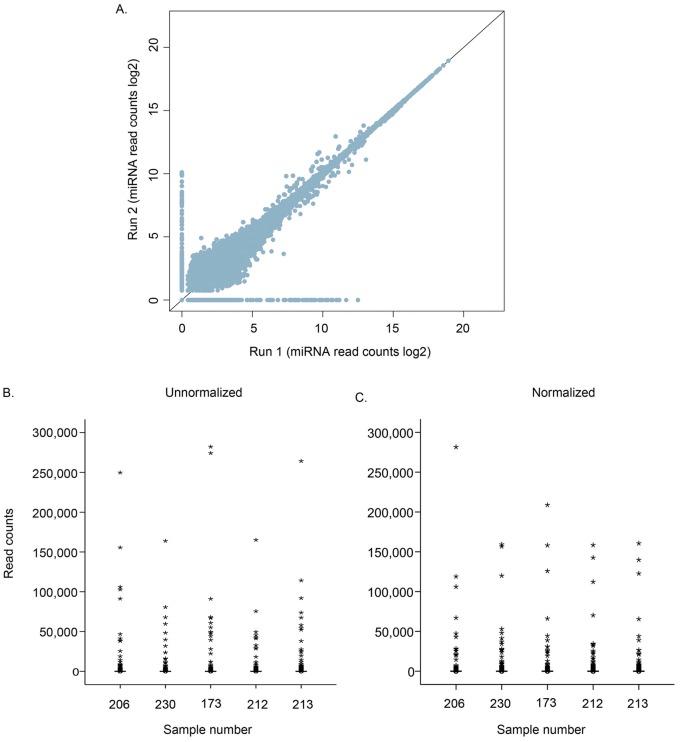
Technical replicates and normalization of miRNA read counts. **A.** Comparison of the technical replicates between run 1 and run 2**.** Each dot represents the total expression of a single miRNA from all the patient samples. The miRNA expression data was normalized and log2 transformed. **B.** Unnormalized miRNA read counts (log2) for five randomly selected patients. **C.** Normalized miRNA read counts (log2) for the same five patients as in B.

In general, to compare expression values between samples it is necessary to normalize against the read count by calculating a normalization factor. The four different normalization methods gave very similar results when comparing the mean change calculated by the difference in percent for each miRNA read count per miRNA (data not shown). Figures 2B and 2C show the distribution of log2 transformed unnormalized read counts and read counts normalized against mature miRNAs for five random patient samples. The lower read counts were not as affected by normalization compared to the higher values. Altogether, normalization generated relatively equally distributed read counts for the majority of miRNAs across all samples.

48 samples were deep sequenced twice, denoted run 1 and run 2, and these data sets were used to compare the results from the separate runs. The results demonstrated a very good correlation between the technical replicates shown by the zero slope line (Figure 2A).

### The Five Most Highly Expressed miRNAs

**Figure 3:**
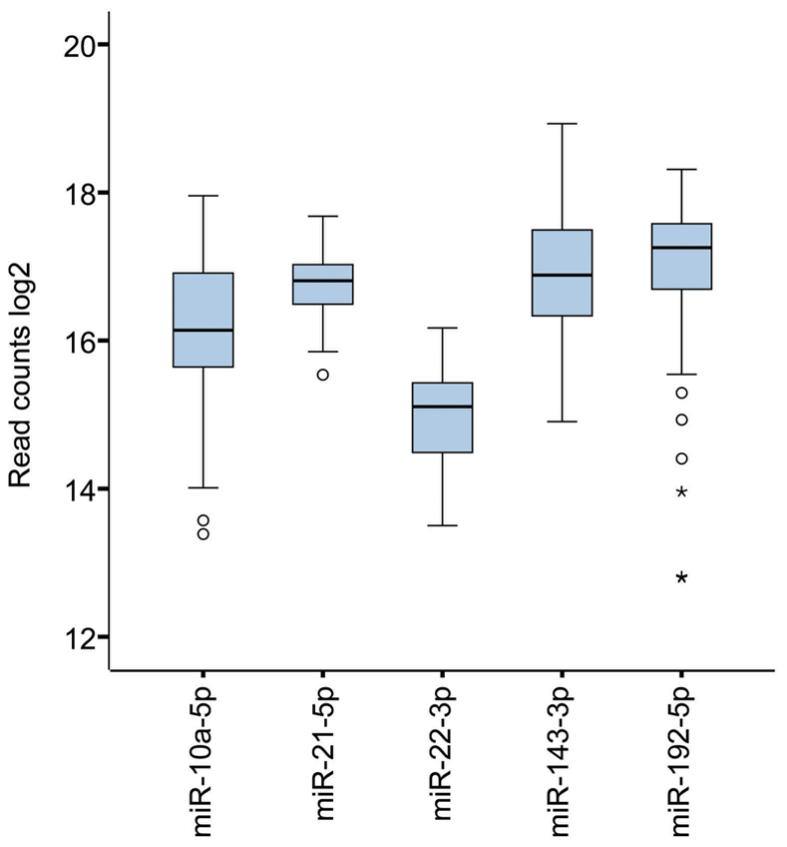
Boxplot of the five most highly expressed miRNAs. Differential expression of the five most abundantly expressed miRNAs in our CRC cohort. The total numbers of miRNA reads are log2 transformed. The circles represent outliers and the stars represent extreme outliers.

The five most abundantly expressed miRNAs in this cohort were miR-10a-5p, miR-21-5p, miR-22-3p, miR-143-3p and miR-192-5p (Figure 3). The read counts for these miRNAs accounted for 53.7% of the total number of miRNA sequences detected in the patient samples, while the top 20 miRNAs accounted for 82.6% of the reads (Table S1). The remaining 503 miRNAs represented 17.4% of the reads. Of the top five miRNA, miR-192-5p had the highest median expression (156,413 reads) while miR-22-3p had the lowest (35,284 reads).

**Figure 4:**
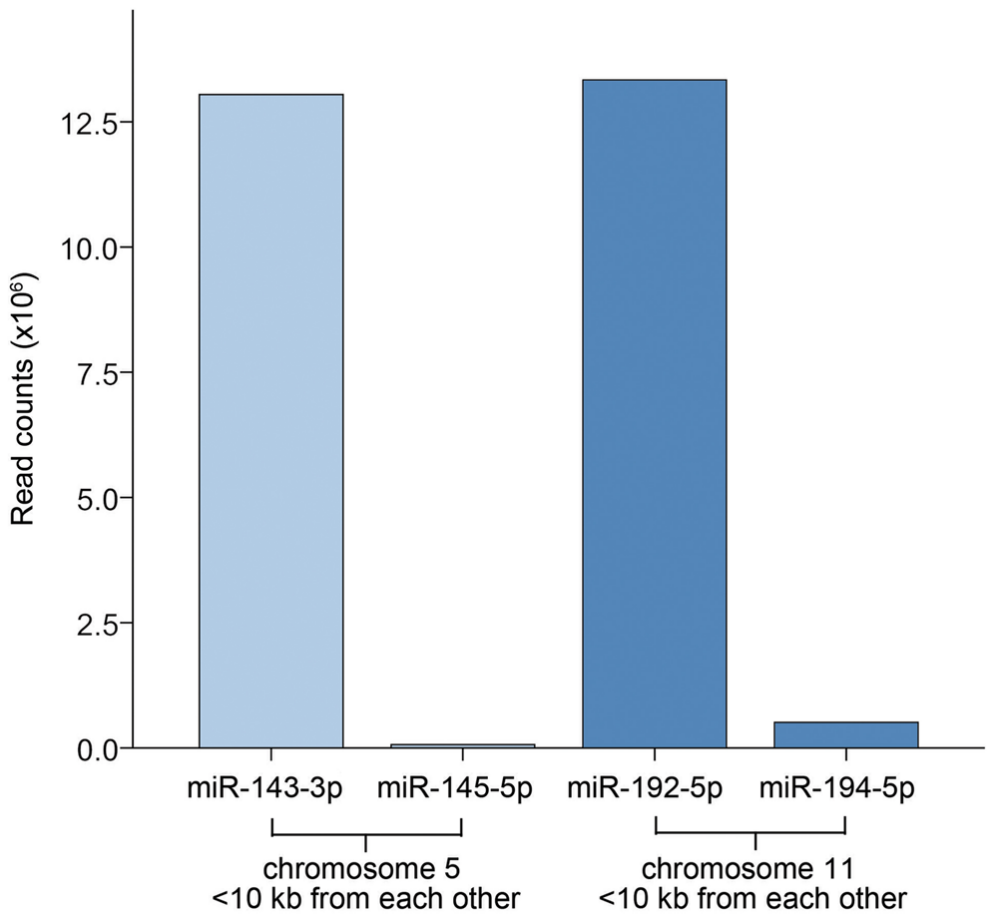
Two of the most highly expressed miRNAs shown with miRNAs from their respective gene clusters. **A.** MiR-143-3p is found in the same gene cluster as miR-145-5p on chromosome 5 (positions 148808481-148808586 and 148810209-148810296, respectively). MiR-192-5p is found in the same gene cluster as miR-194-5p on chromosome 11 (positions 64658609-64658718 and 64658827-64658911, respectively). Although the miRNAs in each gene cluster is <10kb apart, co-expression was not observed.

Many miRNA genes are located in close proximity to other miRNA genes in gene clusters, and two of the top five most highly expressed miRNAs (miR-143-3p and miR-192-5p) are part of such clusters. MiR-143-3p and miR-145-5p are both located on chromosome 5<10kb apart, while miR-192-5p and miR-194-5p are located on chromosome 11<10kb apart. Expression levels of miR-143-3p and miR-192-5p and two miRNAs belonging to their respective gene clusters are depicted in Figure 4. No co-expression was apparent for the miRNAs belonging to these gene clusters, which is in concordance with previous results [29].

### Pathway Analysis for the Five Most Highly Expressed miRNAs

**Figure 5:**
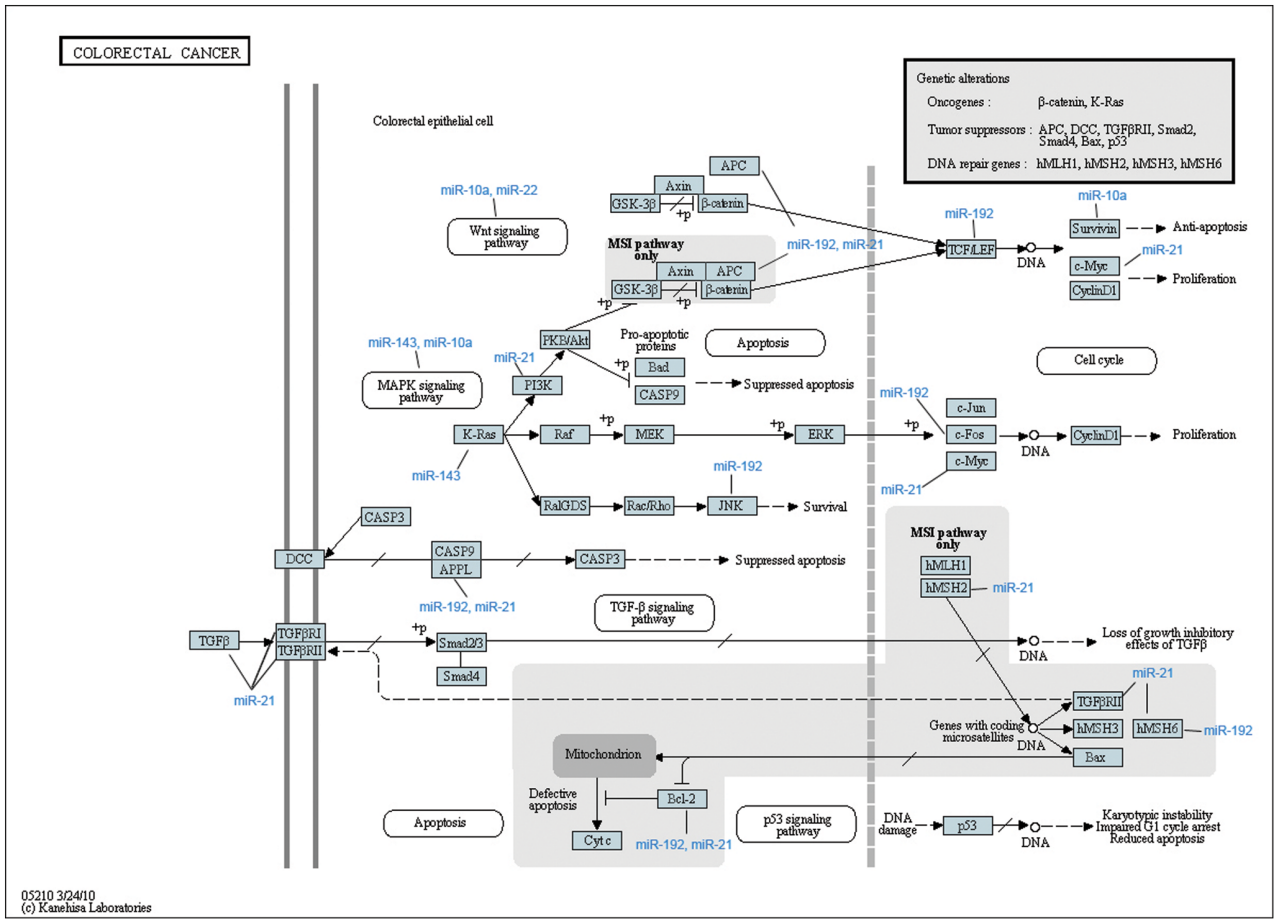
The five most highly expressed miRNAs and the CRC pathway. Pathway analysis results for the target genes of the five most highly expressed miRNAs in the colorectal cancer pathway. The illustration was taken from the KEGG database and the miRNAs were added in blue font to indicate the targets regulated by these miRNAs.

1490 target genes were identified as potentially regulated by miR-10a-5p, miR-21-5p, miR-22-3p, miR-143-3p and miR-192-5p. The pathways with the most significant gene-enrichment are shown in Table 2, and included “Pathways in cancer” (55 genes; p = 3.3×10^−7^), “Cell cycle” (28 genes; p = 2.2×10^−6^), and “Colorectal cancer” (21 genes; p = 1.0×10^−5^). Focusing on the CRC pathway, we found that genes regulated by the five most highly expressed miRNAs included oncogenes (*KRAS, PI3K*), tumor suppressors (APC and TGFβRII), and DNA repair genes (hMSH6) and genes belonging to the wnt and MAPK signaling pathways (Figure 5).

**Table 2:** Pathway analysis of the predicted targets of the five most highly expressed miRNAs.

Term	Number of proteins	p-value
Pathways in cancer	55	3.3×10^−7^
Cell cycle	28	2.2×10^−6^
Colorectal cancer	21	1.0×10^−5^
Pancreatic cancer	19	1.4×10^−5^
Prostate cancer	20	8.4×10^−5^
Bladder cancer	12	4.3×10^−4^
Wnt signaling pathway	26	5.1×10^−4^
Chronic myeloid leukemia	16	9.7×10^−4^
TGF-beta signaling pathway	17	1.7×10^−3^
ErbB signaling pathway	17	1.7×10^−3^

The ten most significant pathways are shown with the number of proteins present in their representative KEGG pathway with respective p-values.

### Correlation between qRT-PCR and Deep Sequencing Data

**Figure 6:**
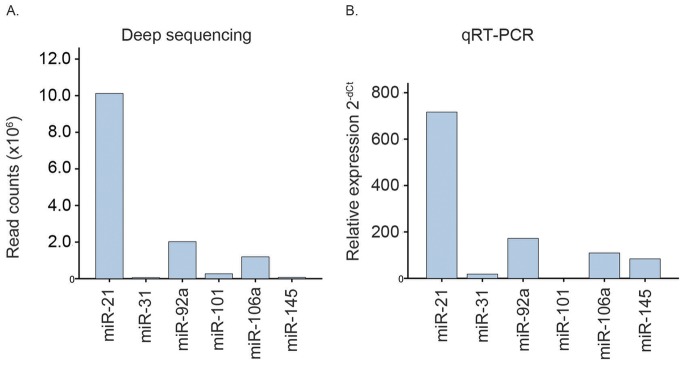
Hierarchical clustering of miRNA expression levels in the CRC cohort. The normalized expression values were log2 transformed and analyzed by unsupervised two-way hierarchical clustering using weighted average linkage (WPGMA). The global map contains all expressed miRNAs shown vertically and the patient samples horizontally.

Expression values for six miRNA miR-21, miR-31, miR-92a, miR-101, miR-106a and miR-145, were previously determined using qRT-PCR [19]. The miRNA expression measured by qRT-PCR was compared to the deep sequencing data using linear regression analysis of normalized Ct values (qRT-PCR) and log2-transformed deep sequencing data. The R^2^ values for the 6 miRNAs tested were 0.06, 0.38, 0.10, 0.001, 0.03, and 0.28 for miR-21, miR-31, miR-92a, miR-101, miR-106a, and miR-145, respectively. The sum of total expression levels was also calculated for these miRNAs for each method, and the relative levels are shown in Figure 6. The relative expression between individual miRNAs was reasonably consistent between methods for four of the miRNAs (miR-21, miR-31, miR-92a and miR-106a), whereas there were clear discrepancies for miR-101 and miR-145. MiR-101 was hardly detectable with qRT-PCR, but exhibited detectable expression values with deep sequencing, while miR-145 was detected by qRT-PCR and hardly detected using deep sequencing.

### Hierarchical Clustering and Associations between miRNA Expression and Clinicopathological Parameters

**Figure 7:**
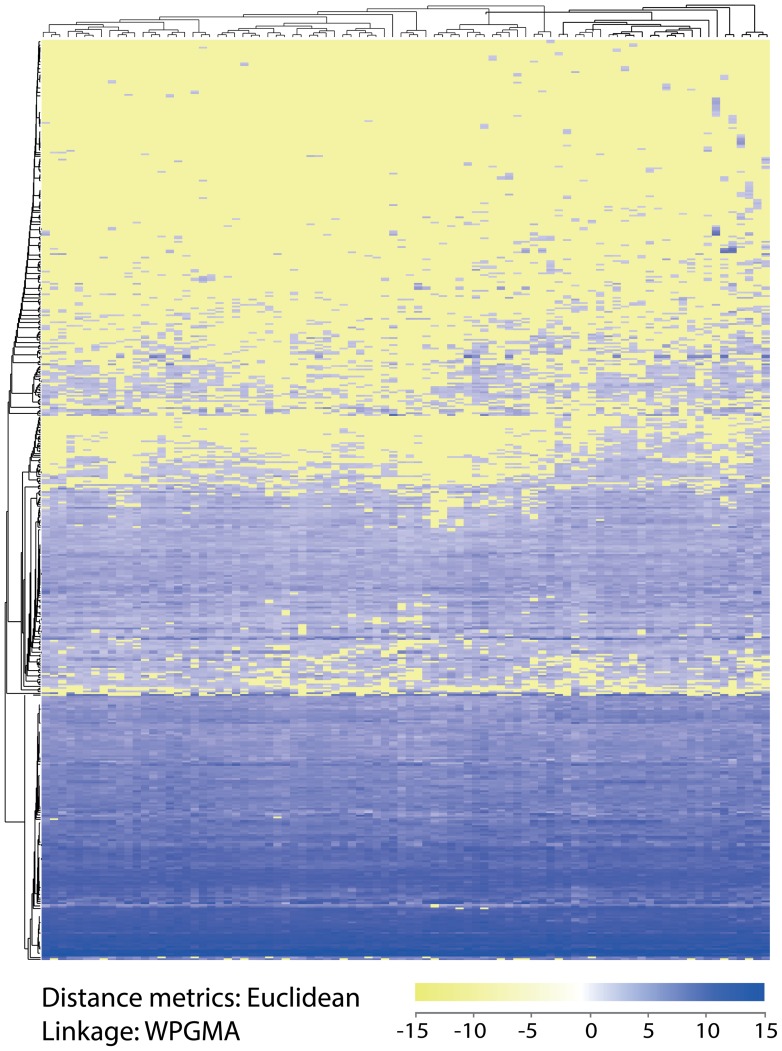
Expression of six selected miRNAs with deep sequencing and qRT-PCR. Expression of six miRNAs (miR-21, miR-31, miR-92a, miR-101, miR-106a and miR-145) is shown from deep sequencing and qRT-PCR for 88 patient samples that were analyzed with both methods. **A.** The bars represent the sum of the total number of reads (x10^6^) for each miRNA from deep sequencing. **B.** The bars represent the sum of the relative expression (2^−dCt^ ) from qRT-PCR.

**Figure 8:**
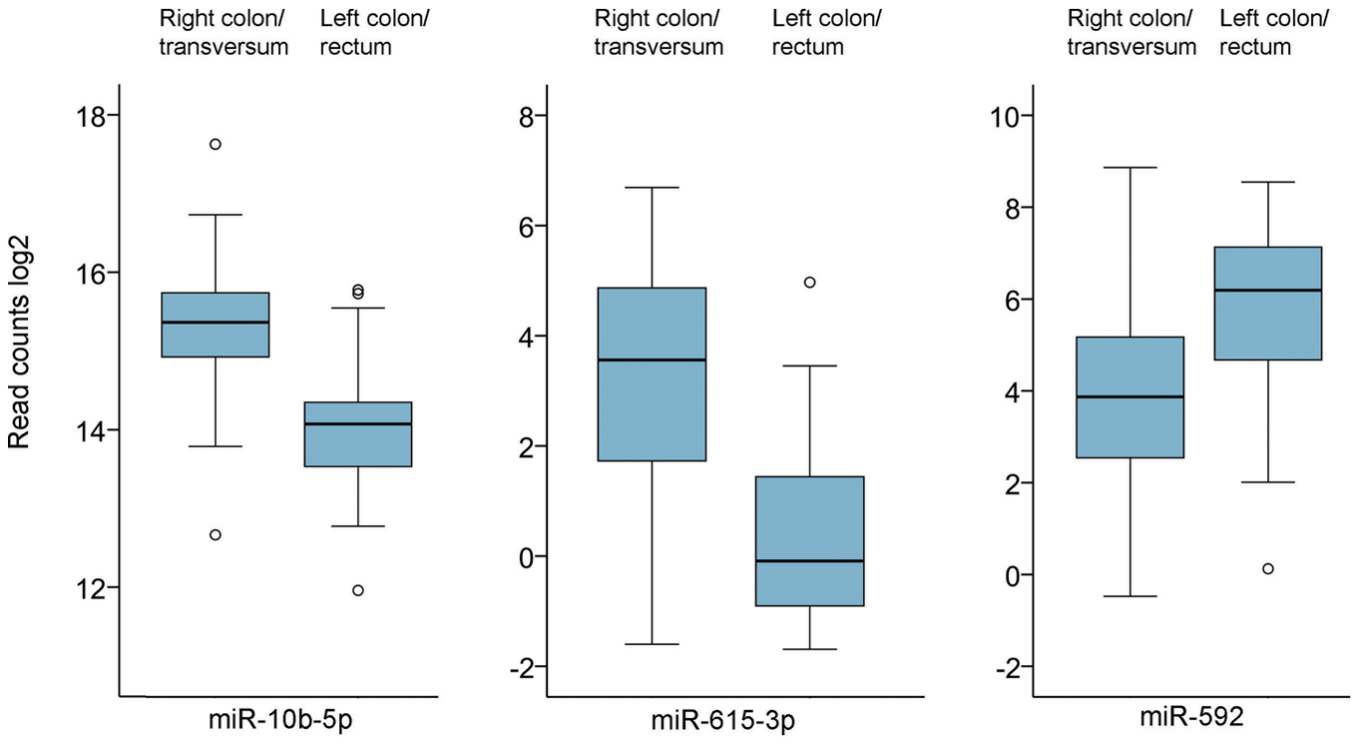
Boxplot of miR-10b-5p, miR-615-3p and miR-592 and tumor localization. High expression of miR-10b-5p and miR-615-3p and low expression of miR-592 were associated with tumor localized in the right colon relative to the left colon and rectum (q <0.001 and p<0.001).

The miRNA expression patterns observed with hierarchical clustering are shown in Figure 7 with miRNAs on the vertical axis and patient samples on the horizontal axis. Most of the miRNAs exhibited very similar expression levels across patient samples. In areas of the plot, some miRNAs appeared to be differentially expressed, but these were almost exclusively located among the miRNA that had very low expression. SAM analysis of expression data and clinicopathological parameters revealed that high expression of miR-10b-5p and miR-615-3p and low expression of miR-592 were associated with tumors located in the right colon (including the ascending and transverse colon) compared to the left colon and rectum (Table 3). The expression of these miRNAs showed 2.4-fold, 41.4-fold and 3.9-fold differences, respectively (q <0.05 for all miRNAs) (Figure 8). High expression of miR-615-3p was also associated with poorly differentiated tumors compared with intermediately and well differentiated tumors (fold change 44.4, q <0.05).

**Table 3:** SAM analysis of tumor localization and differentiation.

	MiRNA	Fold change	FDR	q-value
**Right colon vs. Left colon and rectum**	miR-615-3p	−41.41	<0.05	<0.05
	miR-10b-5p	−2.47	<0.05	<0.05
	miR-592	3.96	<0.05	<0.05
**Differentiation (poor vs. intermediate and well)**	miR-615-3p	−44.41	<0.05	<0.05

Table showing two class unpaired significance analysis with 10000 permutations of the miRNAs which exhibited significant associations with tumor localization and differentiation. FDR: False discovery rate.

### Associations between miRNA Expression and Outcome

In the LASSO and univariate Cox analysis, 5 miRNAs (miR-339-5p, miR-7-1-3p, miR-365b-3p, miR-454-3p, miR-194-3p and miR-15b-3p) with metastasis development as endpoint emerged, and one miRNA (miR-101-5p) with endpoint overall survival was identified, however none of these remained significantly associated with either overall- or metastasis free survival after adjusting for multiple testing. All the miRNAs identified by these analyses were expressed at very low levels in our cohort with the highest median expression observed for miR-454-3p with 187 reads to the lowest for miR-194-3p with only 19 reads.

## Discussion

In the present work, deep sequencing was used to quantify miRNA expression in a large cohort of CRC tumor samples. This approach may contribute potential advantages in global miRNA expression analysis, but also entails new challenges regarding data analysis, as the amount of data collected after deep sequencing contains millions of reads which need to be mapped to the genome and normalized [30]. In the 88 CRC patients successfully analyzed, 523 mature miRNAs were detected. Other small RNA sequences were also detected, but the low detection frequencies of other RNA classes and genomic regions showed that selection for miRNAs had been successful, and in accordance with previous results [29], [31]. In addition, the excellent agreement observed between technical replicates suggested adequate reproducibility.

The five miRNAs most abundantly expressed in the examined CRC cohort were miR-10a-5p, miR-21-5p, miR-22-3p, miR-143-3p and miR-192-5p, and all of these have previously shown to be dysregulated in CRC [32]-[38]. These miRNAs were also among the most highly expressed miRNAs in a previous study performed using deep sequencing of 8 CRC samples and corresponding normal tissues [39]. Interestingly, the top five most highly expressed miRNAs accounted for as much as 54% of the total number of miRNA sequences detected. This is in concordance with a previous deep sequencing study performed on peripheral blood samples, in which the let-7 family accounted for 77% of the total miRNA read counts [29]. The relative importance of high versus low miRNA expression is difficult to interpret, since the absence or abundant presence of miRNAs may represent equally important biological regulatory signals. However, overrepresentation of a small number of miRNAs may imply that these miRNA play important roles as negative regulators of downstream targets and the biological pathways affected by these targets. The predicted targets of the 5 most highly expressed miRNAs were significantly associated with cancer-relevant pathways, including the CRC pathway. Among the predicted targets in the CRC pathway were oncogenes, tumor suppressors and DNA repair genes which are involved in several important signaling pathways including Wnt, MAPK, cell cycle, TGF-β, and p53. Predicted targets (hMSH2 and hMSH6) were also involved with downregulating DNA repair genes affecting microsatellites and are thereby involved in microsatellite instability pathway. These results suggest that the identified top five most highly expressed miRNA are cancer relevant and probably relevant in CRC, but further investigation is necessary to validate the targets and to assess downstream effects.

One of the supposed advantages of deep sequencing compared to microarray analysis and qRT-PCR is improved specificity and sensitivity, suggesting that this method would return more correct measurements of each miRNA than the other methods. When comparing our previous results, measuring expression of six miRNA using qRT-PCR with deep sequencing data, correlation on the individual sample level was poor for all the miRNAs examined. Deep sequencing is often validated by qRT-PCR, but comprehensive comparisons between the two approaches have not been performed. In a deep sequencing study on 9 bladder cancer samples, selected miRNAs from deep sequencing were validated by qRT-PCR, and reported to correlate well when analyzed by fold expression differences in a bar plot [40]. In another study performed on 10 neuroblastoma samples, correlation coefficients between 0.1 and 1 were found when comparing the sum of miRNA expression from qRT-PCR and deep sequencing [41]. The largest discrepancies in our comparison between qRT-PCR and sequencing were found for miR-101 and miR-145. The assay used for qRT-PCR as well as the sequencing were both able to detect but not to separate between known isoforms of these miRNAs. In the sequencing data, no nucleotide variations were found that could explain the discrepancies. In theory, absence of detection of miR-101 by qRT-PCR might be a sensitivity issue, while for miR-145, lack of specificity of the qRT-PCR assay might explain the discrepancies. Thus, it seems unclear whether qRT-PCR can be used for validation purposes, since qRT-PCR and deep sequencing data are generated with different methods and appear on different scales with variable expression ranges, making it difficult to compare the two datasets on a patient-to-patient basis.

One of the aims of this study was to investigate associations between miRNA expression and clinicopathological parameters and outcome. Given the low variability observed between samples, it is not surprising that few such associations were detected. Using univariate Cox proportional hazard regression, no miRNAs were found to be associated with metastasis development or overall survival after correction for multiple testing. No miRNA were selected in the LASSO analysis. Low expression of miR-592 and high expression of miR-10b-5p and miR-615-3p were associated with tumors located in the right colon compared to tumors in the left colon and rectum. High expression of miR-615-3p was also associated with poorly differentiated tumors. MiR-10b was previously reported to be downregulated in CRC and high expression was associated with advanced pT-stage [42], but no such associations were detected in our cohort. MiR-592 has previously been found to be downregulated in tumors with deficient mismatch repair compared to mismatch repair proficient tumors [43], [44]. Deficient mismatch repair gives rise to microsatellite instability, and in sporadic CRC, microsatellite instable tumors are frequently located in the right side of the colon [45], [46]. Thus, low expression of miR-592 in tumors of the right colon in the present study would be consistent with previous findings, but since very little is currently known regarding the targets of this miRNA, clarifying this issue would require further studies. MiR-615 exhibited the most striking difference in expression between the right and left colon in this cohort, and it was also highly expressed in poorly differentiated tumors. There are few reports concerning the expression and function of this miRNA, but in one study, miR-615 expression was downregulated when the proposed tumor suppressor NGX6 was experimentally introduced in the human CRC cell line HT29. In our cohort, 7 of the 10 poorly differentiated tumors were located in the right colon. It remains uncertain which clinicopathological determinant was most important for miR-615 expression, tumor localization or differentiation. Further investigations to determine the relevance of these miRNAs in CRC carcinogenesis and progression are warranted.

Differential expression analysis of miRNAs using deep sequencing has previously been performed in a small cohort of 8 CRC samples [39]. In this study, 37 miRNAs were dysregulated relative to corresponding normal tissue (19 downregulated and 18 upregulated), and 16 miRNA had not previously been reported to be associated with CRC. MiRNA expression in CRC from previous studies have reported quite variable results, both with respect to expression levels and associations with clinicopathological parameters and outcome [47]. Whether these discrepancies result from variations in the methods used, differences between the clinical cohorts analyzed or biological variance are unclear. A contributing explanation to the observed discrepancies may be that many investigators have normalized tumor miRNA expression values against normal samples, using either paired samples or a mixed “normal cohort”, in order to compare samples. The colorectum is a heterogeneously composed organ, consisting of multiple cell types which all may be affected by genetic predispositions and external influences. MiRNA expression in different cell types in the normal colorectum has not been characterized and in addition, the miRNA transcriptome from different “normal” samples has not been extensively compared. The few studies which show miRNA expression in normal colon tissues reveal that the expression is highly variable and in many cases not consistent between individuals [48]. Exploration of miRNA expression in normal tissue and establishing a baseline is needed to establish the relevance of using this normalization strategy.

In the present work, deep sequencing was performed to characterize the miRNA transcriptome of CRC, using tumor samples from a large patient cohort with long-term follow-up. Deep sequencing was technically successful, and a total of 523 mature miRNA were expressed in the samples. Most of the miRNAs exhibited relatively uniform expression between tumor samples, and only few associations were found between expression of specific miRNA and clinical parameters. Specifically, no miRNA emerged as a prognostic biomarker candidate, which in our opinion is in agreement with the highly variable results obtained in similar studies in CRC. The five most highly expressed miRNAs, representing 54% of the detected miRNA sequences, have been predicted to regulate targets involved in cancer pathways, and may represent interesting candidates for future studies of the role of miRNAs in CRC development and progression.

## Supporting Information

Table S1 **Reads detected for the 20 most highly expressed MiRNA, representing 82.6% of all detected reads.**
(DOCX)
