# Supplementary material for: Deep Sequencing the MicroRNA Transcriptome in Colorectal Cancer
Source: PLoS One. 2013 Jun 18;8(6):e66165. doi: 10.1371/journal.pone.0066165 (PMC3688869; doi:10.1371/journal.pone.0066165)
Supplement: Table S1 — Reads detected for the 20 most highly expressed MiRNA, representing 82.6% of all detected reads. (DOCX) [file pone.0066165.s001.docx]

Supplementary Table 1

Reads detected for the 20 most highly expressed MiRNA, representing 82.6 % of all detected reads.

| miRNA_ID | Sum | Mean | Median | Min | Max |
| --- | --- | --- | --- | --- | --- |
| hsa-miR-192-5p | 13338186 | 149867 | 156413 | 7120 | 325278 |
| hsa-miR-143-3p | 13047274 | 146599 | 120932 | 30724 | 499304 |
| hsa-miR-21-5p | 10122907 | 113741 | 114814 | 47580 | 210017 |
| hsa-miR-10a-5p | 8180779 | 91919 | 72255 | 10718 | 254012 |
| hsa-miR-22-3p | 3052128 | 34294 | 35284 | 11605 | 73839 |
| hsa-miR-148a-3p | 3018613 | 33917 | 30887 | 3927 | 124102 |
| hsa-miR-10b-5p | 2883037 | 32394 | 23445 | 3998 | 203299 |
| hsa-miR-26a-5p | 2880190 | 32362 | 30594 | 16728 | 58930 |
| hsa-let-7a-5p | 2748907 | 30887 | 30658 | 15152 | 56944 |
| hsa-let-7f-5p | 2104406 | 23645 | 22775 | 10295 | 41887 |
| hsa-miR-92a-3p | 2025529 | 22759 | 20061 | 5624 | 68188 |
| hsa-miR-27b-3p | 1945191 | 21856 | 19804 | 9229 | 62257 |
| hsa-miR-181a-5p | 1944863 | 21852 | 19077 | 0 | 109152 |
| hsa-miR-191-5p | 1188307 | 13352 | 12399 | 0 | 30877 |
| hsa-miR-182-5p | 1123631 | 12625 | 10099 | 2041 | 44928 |
| hsa-miR-30d-5p | 992774 | 11155 | 9895 | 5073 | 36406 |
| hsa-miR-30e-5p | 764734 | 8593 | 8096 | 4438 | 15996 |
| hsa-miR-200b-3p | 703712 | 7907 | 6136 | 0 | 43322 |
| hsa-let-7g-5p | 688477 | 7736 | 7574 | 0 | 14775 |
| hsa-miR-146b-5p | 665601 | 7479 | 6272 | 1043 | 48809 |
